# Impact of rare and low-frequency sequence variants on reliability of genomic prediction in dairy cattle

**DOI:** 10.1186/s12711-018-0432-8

**Published:** 2018-11-20

**Authors:** Qianqian Zhang, Goutam Sahana, Guosheng Su, Bernt Guldbrandtsen, Mogens Sandø Lund, Mario P. L. Calus

**Affiliations:** 1 Department of Molecular Biology and Genetics, Center for Quantitative Genetics and Genomics, Aarhus University, Tjele, Denmark; 2 Wageningen University and Research, Animal Breeding and Genomics, Wageningen, The Netherlands; 3 Present Address: Department of Veterinary and Animal Sciences, Faculty of Health and Medical Sciences, University of Copenhagen, Copenhagen, Denmark

## Abstract

**Background:**

Availability of whole-genome sequence data for a large number of cattle and efficient imputation methodologies open a new opportunity to include rare and low-frequency variants (RLFV) in genomic prediction in dairy cattle. The objective of this study was to examine the impact of including RLFV that are within genes and selected from whole-genome sequence variants, on the reliability of genomic prediction for fertility, health and longevity in dairy cattle.

**Results:**

All genic RLFV with a minor allele frequency lower than 0.05 were extracted from imputed sequence data and subsets were created using different strategies. These subsets were subsequently combined with Illumina 50 k single nucleotide polymorphism (SNP) data and used for genomic prediction. Reliability of prediction obtained by using 50 k SNP data alone was used as reference value and absolute changes in reliabilities are referred to as changes in percentage points. Adding a component that included either all the genic or a subset of selected RLFV into the model in addition to the 50 k component changed the reliability of predictions by − 2.2 to 1.1%, i.e. hardly no change in reliability of prediction was found, regardless of how the RLFV were selected. In addition to these empirical analyses, a simulation study was performed to evaluate the potential impact of adding RLFV in the model on the reliability of prediction. Three sets of causal RLFV (containing 21,468, 1348 and 235 RLFV) that were randomly selected from different numbers of genes were generated and accounted for 10% additional genetic variance of the estimated variance explained by the 50 k SNPs. When genic RLFV based on mapping results were included in the prediction model, reliabilities improved by up to 4.0% and when the causal RLFV were included they improved by up to 6.8%.

**Conclusions:**

Using selected RLFV from whole-genome sequence data had only a small impact on the empirical reliability of genomic prediction in dairy cattle. Our simulations revealed that for sequence data to bring a benefit, the key is to identify causal RLFV.

**Electronic supplementary material:**

The online version of this article (10.1186/s12711-018-0432-8) contains supplementary material, which is available to authorized users.

## Background

Due to the progress in sequencing technology, whole-genome sequence data has become available for large numbers of dairy cattle [1–3]. Using this resource, individuals with single nucleotide polymorphism (SNP) chip genotype data can be imputed to whole-genome sequence variants [4, 5]. Causal variants are expected to be better identified by using whole-genome sequence data, and therefore, can be used to improve the reliability of genomic prediction [6]. Brondum et al. [7] found that the reliability of genomic prediction increased by up to 4% and 0.5% for milk yield traits and fertility, respectively, when quantitative trait loci (QTL) derived from whole-genome sequence data were used. However, in dairy cattle, van Binsbergen et al. [8] observed no increase in reliability of prediction when all imputed whole-genome sequence variants compared to only the QTL derived from sequence data were used.

The SNP chips that are routinely used in genomic prediction in dairy cattle include mostly SNPs with a relatively high minor allele frequency (MAF) that can efficiently tag common variants. However, the ability of these chips to tag rare and low-frequency variants (RLFV) is limited. There are several indications that RLFV make an important contribution to genetic variance. For instance, nonsynonymous SNPs are expected to make a larger contribution to genetic variance than synonymous SNPs [9]. In the human genome, non-synonymous variants tend to have a lower MAF than synonymous variants [10]. More generally, functional variants are much more likely to be rare than common variants [11]. Rare variants are more often deleterious variants and, therefore, more prone to being purged from the population. Therefore, including RLFV in genomic prediction, through (imputed) sequence data, might increase the reliability of genomic prediction. This may be especially the case for fitness traits, since alleles with deleterious effects are expected to strongly affect fitness and to be rare due to purging from the population. To date, the question about whether the inclusion of subsets of RLFV from imputed whole-genome sequence that are selected by different strategies, in addition to e.g. 50 k SNP chip data, improves the reliability of genomic prediction in dairy cattle has not been investigated.

There are various ways to select a subset of RLFV from whole-genome sequence for inclusion in genomic prediction [12, 13]. One approach is to perform a genome-wide association study (GWAS) for RLFV, which allows to select those that are significantly associated with the trait of interest, under the condition that the existing sample size and the effect of the RLFV are large enough to be identified. Although the effect of RLFV is expected to be small, the power to detect RLFV can be improved when combining RLFV within a gene or region. The power to detect causal RLFV can be considerably increased by using methods that are specifically designed to map RLFV instead of the commonly used mixed linear models, as shown in a simulation study in dairy cattle [14]. In addition, for fitness-related traits, variants with a lower MAF are expected to have a larger effect [15], which suggests that for genomic prediction of fitness-related traits, RLFV may be more relevant, in spite of their low MAF. An alternative approach is to use the annotation of variants that predict the biological impact of variants in general [16–18]. It can be hypothesized that RLFV with high impact annotations, e.g. protein altering variants, probably have a larger effect on phenotypes, and therefore should be included in genomic prediction. Gonzalez-Recio et al. [19] explored the contribution of rare variants in the genetic variance of milk-related traits and fertility, and examined the accuracy of genomic prediction using all the rare variants from transcripts for these traits. Their study and our previous data [20] suggested that the relative contribution of RLFV to the total genetic variance might be somewhat higher for health-related traits such as fertility, disease susceptibility and longevity than for milk production traits. Therefore, in this study, we made the hypothesis that the reliability of genomic prediction can be increased by including selected subsets of RLFV for three fitness-related indices, i.e. fertility, health, and longevity, in dairy cattle.

The objective of this study was to test the above hypothesis and to empirically examine the impact of using selected RLFV from imputed whole-genome sequence data on the reliability of genomic prediction in dairy cattle. We also undertook a simulation study to evaluate what are the critical factors that increase the reliability of genomic prediction as a result of using RLFV in the model, depending on the amount of genetic variance explained by causal RLFV.

## Methods

### Phenotypes and genotypes

In total, 6337 Holstein sires with de-regressed proofs (DRP) were genotyped using the Illumina BovineSNP50 BeadChip (50 k) version 1 or 2 (Illumina Inc., San Diego, CA) [21]. The following quality criteria were applied for selecting SNPs: minimum call rates were set to 85% for individuals and 95% for SNPs, and monomorphic SNPs or deviating from Hardy–Weinberg proportions (P < 0.00001) were excluded. Thus, 43,415 autosomal SNPs remained for the analyses. The genome position of the SNPs were based on the UMD3.1 Bovine genome assembly [22]. The 50 k genotypes of the 6337 animals were imputed to the sequence level by a two-step approach that was developed by Brondum et al. [4]. They showed that this strategy yields the highest imputation accuracy with optimal computation time for this particular type of data. The bulls’ 50 k genotypes were first imputed to a high-density SNP array (HD, 734,077 SNPs) using a multi-breed reference of 3383 animals, which were genotyped with the Illumina BovineHD chip (Illumina Inc., San Diego, CA) by using IMPUTE2 software [23]. These imputed HD genotypes were subsequently imputed to the whole-genome sequence level with 22,232,889 variants (SNPs, short insertions and deletions) by using a multi-breed reference of 1228 animals from *run4* of the 1000 bull genomes project [24] and from Aarhus University [25–27]. The reference genotype probabilities that had an imputation quality (the R^2^ value) at imputed markers lower than 0.9 were removed from the original sequence data [4]. Both the 50 k and the whole-genome sequence genotypes were pre-phased with BEAGLE v3.3.2 [28]. The subsequent imputation step was performed using the Minimac2 software [29]. Imputed variants with a MAF lower than 0.001 were filtered out, which removed SNPs that had less than 13 copies of the minor allele from the data, considering that the total number of alleles is equal to twice the number of animals (6337). The average imputation accuracy (the estimated value of the squared correlation between imputed genotypes and true genotypes from Minimac2) was equal to 0.850 with a standard deviation of 0.233 for rare variants (MAF < 0.01) and 0.873 with a standard deviation of 0.215 for low-frequency variants (0.01 < MAF < 0.05).

All called variants were annotated using Variant Effect Predictor (VEP) and ENSEMBL (v.67) databases [30]. Any sites with multiple transcripts that resulted in multiple annotations were annotated only once using the *by*-*gene* option in VEP [30] and annotations of the non-reference alleles were classified, as explained later, according to SIFT scores of the variants [31]. VEP determines the effect of the variants (SNPs, short insertions and deletions) on genes, transcripts, and protein sequence. SIFT predicts the potential effect that a non-reference allele has on encoded proteins, and integrates the effect of amino acid change, folding structure (predicted or known), and conservation score [32].

### Selection of RLFV from imputed sequence data

RLFV were defined as the imputed sequence variants that had a MAF lower than 0.05 and were selected based on their genic or intergenic localization, annotation and association mapping. Depending on the amino acid change that results from having the rare instead of the alternative allele, rare variants are assigned to a different class of annotations (see classifications below). We selected four sets of RLFV in this study:

1. RLFV in all 23,431 genes including non-coding genes;
2. RLFV with annotations of ‘high impact’ from VEP and SIFT scores (frameshift variant, inframe deletion, inframe insertion, missense variant, protein altering variant, start lost, stop gained, stop lost, splice acceptor variant, splice donor variant, splice region variant) according to how much the amino acid can be changed;
3. RLFV from set (2) in addition to those with annotations of ‘medium impact’ (3 prime UTR variant, 5 prime UTR variant, downstream gene variant, synonymous variant, upstream gene variant) according to how much the amino acids can be changed;
4. RLFV in genes that were associated with the analyzed index trait by considering only the individuals included in the training population using the MONSTER software [33]. The details for mapping genic RLFV by the famSKAT approach [34] are described in Zhang et al. [14]. Briefly, famSKAT models the effects of all genic RLFV in a genetic region such as a gene, as random effects with the same variance, without making any assumption about the direction of the effects of the genic RLFV across the loci considered. The genes with a *p* value lower than 0.01 from famSKAT approach were selected and the RLFV within these genes were extracted. Briefly, the model is:
  $${\mathbf{y}} = {\mathbf{X}}{\varvec{\upgamma}} + {\mathbf{Zu}} + {\mathbf{M}}{\varvec{\upbeta}} + {\mathbf{e}},$$
  where ${\mathbf{y}}$ is a vector of phenotypes, ${\mathbf{X}}$ is a design matrix for fixed covariates including the intercept, ${\varvec{\upgamma}}$ is a vector of unknown covariate effects, ${\mathbf{Z}}$ is an incidence matrix relating phenotypes to the corresponding random polygenic effect, ${\mathbf{u}}$ is a vector of random polygenic effects that follows a multivariate normal distribution $N(0,{\mathbf{A}}\sigma_{a}^{2}$), where ${\mathbf{A}}$ is the pedigree-based additive genetic relationship matrix and $\sigma_{a}^{2}$ is the polygenic variance, ${\mathbf{e}}$ is a vector of random residuals, ${\mathbf{e}}\sim\,N\left( {0,{\mathbf{I}}\sigma_{e}^{2} } \right)$, ${\mathbf{M}}$ is a $n \times m$ matrix that encodes the genotype at the $m$ tested variant loci and $n$ is the number of individuals with $m_{ij}$ representing allele dosage (0, 1 or 2) of the minor allele at the $j$th variant of individual $i$, and ${\varvec{\upbeta}}$ is a vector of random effects of the $m$ variants, ${\varvec{\upbeta}}\sim\,N\left( {0,{\mathbf{I}}\sigma_{q}^{2} } \right)$.

Finally, we examined a scenario that randomly sampled a similar number of RLFV when RLFV with medium-to-high impact annotations were included in the prediction model. This analysis was done for the health index because it showed the largest improvement in reliability of prediction compared to when 50 k SNPs were used in the prediction model.

Three fitness-related index traits (fertility, health and longevity) were studied, for which the phenotypes were DRP. In total, 5043, 4926 and 4673 bulls with DRP, imputed sequence data, 50 k data and pedigree information were available for fertility, health and longevity, respectively. The fertility index is a linear combination of breeding values for interval from first to last insemination, number of inseminations for heifers and cows and interval from calving to first insemination for cows. The health index is a linear combination of breeding values for diseases other than clinical mastitis, which describe the genetic risk of reproductive, metabolic and feet-and-leg diseases. These breeding values are based on recorded treatments by veterinarians during the first three lactations. The longevity index describes the genetic potential for the cows’ productive longevity. For details regarding the index traits, recording procedures and models used to estimate their breeding values, see http://www.nordicebv.info/ntm-and-breeding-values.

### Simulation of causal RLFV

Simulations were undertaken to evaluate the (potential) impact of including selected RLFV in the prediction model on the reliability of genomic prediction, assuming that rare causal variants explained a small proportion of the genetic variance. In these simulations, quantitative trait nucleotides (QTN) were simulated by randomly drawing RLFV from genes based on the assumption that the power of detecting RLFV can be improved when combining RLFV, each with a supposedly small effect, within a gene or genomic region. Three scenarios were simulated:

1. SQTN (QTN simulated with a small effect): seven to ten genes were randomly selected per chromosome and the RLFV from these genes were simulated as QTN.
2. MQTN (QTN simulated with a medium effect): one gene per chromosome was randomly selected and the RLFV within these genes were simulated as QTN.
3. LQTN (QTN simulated with a large effect): nine genes were randomly selected across the whole genome and the RLFV in these genes were simulated as QTN.

From scenario (1) to (3), the number of simulated QTN decreased, while the variance explained by each QTN increased. Therefore, we expected a gradual increase in the power of detection of the simulated rare QTN from scenario (1) to (3).

Effects for the rare QTN were sampled from a normal distribution $N\varvec{ }\sim \left( {0, 1} \right)$. The true breeding values for the simulated rare QTN for all individuals [denoted as a column vector (${\mathbf{TBV}}_{{\mathbf{Q}}}$)] were calculated as:

$${\mathbf{TBV}}_{{\mathbf{Q}}} = {\mathbf{M}}_{{\mathbf{Q}}} \times {\varvec{\upalpha}},$$

where ${\mathbf{M}}_{{\mathbf{Q}}}$ is the genotype matrix including the rare QTN (one row per animal), and **α** is the column vector of QTN effects. Then, the values in ${\mathbf{TBV}}_{{\mathbf{Q}}}$ were scaled such that the variance jointly explained by the rare QTN was equal to 10% of the estimated genetic variance explained by the SNPs on the 50 k chip. Finally, the scaled ${\text{TBV}}_{\text{Q}}$ for each individual was added to the fertility DRP to obtain the simulated phenotypes i.e. ${\text{y}} = {\text{DRP}} + {\text{TBV}}_{\text{Q}}$, where ${\text{y}}$ is the simulated phenotype and DRP is the observed phenotype. The total variance explained by these simulated QTN was equal to 10% of the estimated genetic variance explained by the markers on the 50 k chip, i.e. 14.5. Thus, for the simulated data, a heritability of 0.485 (i.e. (14.5 + 145)/328.8) was obtained and used, since the observed heritability for fertility was 0.441.

For each of the three simulation scenarios, we tested three strategies for selecting RLFV to include in the subsequent genomic prediction step:

1. Genotypes of the simulated QTN were used to compute the second GRM used in prediction model, and thus we assumed that the QTN were known without error.
2. The RLFV from 10 randomly selected genes per chromosome were added to the simulated QTN to construct the second GRM in the prediction model, with none of these variants having a simulated effect. The second strategy mimicked real situations more closely, i.e. in which false positive associations add noise in the prediction model.
3. The RLFV were selected based on significance of association mapping (see subset (4) in “Selection of RLFV from imputed sequence data” section).

Each simulation scenario was replicated 10 times, thus the reported reliabilities and the measure of bias estimated by the slope of the regression of the sum of DRP and ${\text{TBV}}_{\text{Q}}$ on genomic estimated breeding values (GEBV) were averaged across replicates. Standard errors of these average values were calculated as the standard deviation of the results across the 10 replicates divided by $\sqrt {10}$. Due to computational limitations, the simulations were undertaken only for fertility, and the rare variant association mapping (second strategy) was applied only to one randomly selected replicate.

### Genomic prediction

The GBLUP model was used to predict genomic breeding values using the following model:

1 $${\mathbf{y}} = {\textbf{1}}^{{\prime }} \mu + {\mathbf{Z}}_{{\mathbf{g}}} {\mathbf{g}} + {\mathbf{e}},$$

where ${\mathbf{y}}$ is the vector of phenotypes, ${\textbf{1}}$ is a vector of 1s, $\mu$ is the general mean, and ${\mathbf{Z}}_{{\mathbf{g}}}$ is the design matrix which allocates ${\mathbf{y}}$ to ${\mathbf{g}}$. Vector ${\mathbf{g}}$ contains random additive genetic effects with a variance of ${\mathbf{G}}\sigma_{{\mathbf{g}}}^{2}$, where ${\mathbf{G}}$ is the genomic relationship matrix (GRM) calculated following VanRaden’s method 1 [35]:

$${\mathbf{G}} = \frac{{\left( {{\mathbf{X}} - 2{\mathbf{p}}1^{{\prime }} } \right)\left( {{\mathbf{X}} - 2{\mathbf{p}}1^{{\prime }} } \right)'}}{{2\mathop \sum \nolimits_{j - 1}^{n} p_{j} \left( {1 - p_{j} } \right)}},$$

where ${\mathbf{X}}$ is the allele sharing matrix with the number of copies of the second allele. ${\mathbf{p}}$ is a vector with allele frequencies ($p_{j}$ is the frequency of the second allele at locus $j$) and ${\textbf{1}}$ is a vector of 1s. The factor $2\sum\nolimits_{j - 1}^{n} {p_{j} (1 - p_{j} )}$ scales ${\mathbf{G}}$ to be comparable to the pedigree-based relationship matrix.

An alternative GBLUP model is:

2 $${\mathbf{y}} = {\textbf{1}}^{{\prime }} \mu + {\mathbf{Z}}_{{{\mathbf{g}}_{1} }} {\mathbf{g}}_{1} + {\mathbf{Z}}_{{{\mathbf{g}}_{2} }} {\mathbf{g}}_{2} + {\mathbf{e}},$$

which is used in the analysis when two GRM were fitted simultaneously. The symbols in Model (2) were the same as in Model (1). The vectors ${\mathbf{g}}_{1}$ and ${\mathbf{g}}_{2}$ were the two random additive genetic effects corresponding to the two GRM. The two GRM are computed with the same formula as in Model (1).

Model (1) with the GRM built using 50 k genotype data was used as the reference model for comparisons with other approaches. Genomic prediction with selected RLFV were done using Model (2) in which the first GRM was based on 50 k data and the second GRM was built using the RLFV selected by one of the strategies as described above. Estimation of variance components and prediction of breeding values were carried out using the DMU software [36].

For each index trait, the 1000 youngest bulls with a birth date between 23/07/2005 and 14/01/2009 were used as validation bulls. The remaining bulls (4043 for fertility, 3926 for health and 3673 for longevity) born before 23/07/2005 were used as training data. The reliability of genomic prediction was measured as the squared correlation between GEBV and DRP divided by the mean reliability of DRP for validation individuals, i.e. $r_{\text{GEBV}}^{2} = ({\text{cor}}({\text{GEBV}},{\text{DRP}}))^{2} /\bar{r}_{\text{DRP}}^{2}$. Standard errors for the accuracies, i.e. the square roots of the reliabilities, were approximated using ${{\left( {\frac{{\left( {1 - r_{\text{GEBV}}^{2} } \right)}}{{\sqrt {N - 2} }}} \right)} \mathord{\left/ {\vphantom {{\left( {\frac{{\left( {1 - r_{\text{GEBV}}^{2} } \right)}}{{\sqrt {N - 2} }}} \right)} {\bar{r}_{\text{DRP}} }}} \right. \kern-0pt} {\bar{r}_{\text{DRP}} }}$, where *N* is the size of the training data [37]. A measure of bias was estimated by the slope of the regression of DRP on GEBV in the validation dataset: i.e. $\hat{b} = \frac{{{\text{cov}}\left( {{\text{DRP}},{\text{GEBV}}} \right)}}{{{\text{var}}\left( {\text{GEBV}} \right)}}$. Any deviation from 1 in this regression slope is an indication of bias of the scale of the GEBV.

## Results

### Impact of including RLFV on the reliability of genomic prediction

The total number of genic RLFV ranged from 1,585,116 to 1,605,553 across the three index traits (see Additional file 1: Table S1). Based on the association test, 0.4–1.2% genic RLFV of the total number of RLFV were selected across the three index traits. Less than 1% of the RLFV had high impact annotations, while less than 3% of the total RLFV had medium-to-high impact annotations (see Additional file 1: Table S1). The reliabilities of genomic prediction obtained by using different sets of selected RLFV are in Table 1. The reliability of prediction obtained with the 50 k data was used as the reference scenario to compare alternative scenarios for each index trait. Reliabilities are presented on a scale from 0 to 100%, and hereafter absolute changes in reliabilities, i.e. changes in percentage points, are reported. Adding an additional component with either all genic or a subset of selected RLFV into the model to the 50 k component changed the reliability by − 2.2 to 1.1%. Thus, on average the prediction reliability hardly changed, regardless of how the RLFV were selected (Table 1). For the scenario that showed the largest improvement in reliability of prediction (i.e. 0.7% obtained by adding RLFV with medium-to-high impact annotations for the health index), we tested whether the improvement of the reliability of prediction was the result of simply increasing the number of RLFV in the model. We observed that adding a similar number of randomly selected RLFV increased reliability on average by 0.6% across 10 different random subsets. This suggests that the observed small improvement for the health index is due to the addition of a considerable number of genic RLFV, and not necessarily to the addition of RLFV with medium-to-high impact annotations. When RLFV with high impact annotations were added as an additional genetic component, the reliability of prediction decreased for all three index traits. To gain more insight into these observations, we compared the estimates of variance components using different marker sets in the prediction models (Table 2). For all three index traits, the variance explained by the RLFV was largest when all genic RLFV were included in the prediction model, followed by the scenarios with RLFV with medium and high impact annotations, while the genic RLFV selected by association mapping always explained the smallest genetic variance.

**Table 1:** Reliability of genomic prediction using different marker sets

Marker sets	Fertility	Health	Longevity
50 k SNP array	39.2	31.9	28.5
50 k + All genic RLFV	40.3	32.6	27.7
50 k + RLFV in genes with significant association	39.9	31.8	27.8
50 k + RLFV with medium-to-high impact annotations	39.7	32.6	27.1
50 k + RLFV with high impact annotations	38.4	31.5	26.3

Reliabilities are presented on a scale from 0 to 100%. Accuracies, i.e. the square roots of the reliabilities, ranged from 0.513 to 0.635 on a scale from 0 to 1, while the corresponding standard errors ranged from 0.011 to 0.014

**Table 2:** Estimates of additive genetic variance from different models using various marker sets for fertility, health and longevity

Marker sets/index traits	Average number of variants	Fertility		Health		Longevity	
Variance components		50 k	RLFV	50 k	RLFV	50 k	RLFV
50 k	54,323	144.5	–	146.6	–	142.2	–
50 k + all genic RLFV	1,650,799	113.2	27.0	119.2	25.1	95.2	42.9
50 k + RLFV in genes with significant association	191,414	139.2	11.0	142.9	6.0	115.9	26.5
50 k + RLFV with medium-to-high impact annotations	577,732	117.7	22.2	120.3	23.5	97.7	39.8
50 k + RLFV with high impact annotations	81,717	131.6	17.0	123.2	21.4	105.8	31.9

We observed that the bias of the GEBV measured by regression slope for health and longevity was reduced by adding increasingly more RLFV, regardless of how they were selected (Table 3). However, the bias of the GEBV measured by regression slope was increased for fertility when adding increasingly more RLFV (Table 3). Across all three index traits, these results show that adding increasingly more RLFV, leads to an increasingly larger variance of the GEBV.

**Table 3:** Bias of the GEBV measured by regression slope in different methods of selection of rare and low-frequency variants (RLFV)

Methods of selection of RLFV	Fertility	Health	Longevity
50 k SNP array	0.993	0.902	0.851
50 k + all genic RLFV	1.046	0.950	0.939
50 k + RLFV in genes with significant association	1.012	0.913	0.925
50 k + RLFV with medium-to-high impact annotations	1.040	0.956	0.931
50 k + RLFV with high impact annotations	1.038	0.935	0.902

### RLFV simulated as QTN

To validate the potential impact of including rare causal variants in the model on the reliability of genomic prediction, we simulated genic RLFV as QTN and re-estimated the reliabilities of prediction using similar strategies to select RLFV used for prediction. In our study, only one replicate was randomly selected from each simulation scenario (Tables 4, 5 and 6), because computational limitations prohibited the mapping of genic RLFV for all 10 replicates. Results across all 10 replicates for all scenarios, except that based on association mapping of genic RLFV, are in Tables S2, S3 and S4 (see Additional file 2: Tables S2, Additional file 3: S3, Additional file 4: S4).

**Table 4:** Characteristics for one random replicate of each simulation scenario

Characteristics	SQTN	MQTN	LQTN
Number of genes	7–10 per chr	1 per chr	9
Number of genic RLFV simulated as QTN	21,468	1348	235
Number of RLFV from 10 random selected genes from each chromosome	29,166	27,290	28,308
Number of RLFV from mapped genes	78,080	81,010	80,999

Chr = chromosome

RLFV refer to rare and low-frequency variants and QTN refer to quantitative trait nucleotides. SQTN corresponds to the scenario with RLFV in seven to ten genes per chromosome simulated as causal variants; MQTN corresponds to the scenario with RLFV in one gene per chromosome simulated as causal variants; LQTN corresponds to the scenario with RLFV in nine randomly selected genes across the whole genome simulated as causal variants. The simulated total variances for the QTN in SQTN, MQTN and LQTN were 10% of the estimate of variance explained by 50 k markers for fertility index

The numbers of selected genic RLFV in the different scenarios from one replicate are in Table 4. The number of genic RLFV simulated as QTN ranged from 235 to 21,468. When all simulated QTN were included with the 50 k SNPs, the prediction reliabilities improved from 3.4% for the MQTN simulation scenario to 6.8% for the SQTN simulation scenario (Table 5). Adding randomly selected genic RLFV (without a simulated effect) to the simulated QTN, decreased the reliability from 1.2% in the SQTN simulation scenario to 4.3% in the LQTN simulation scenario compared with all simulated QTN included in the prediction model (Table 5). Across all simulation scenarios, adding genic RLFV from significant association mapping improved the reliability of prediction from 1.3% for the MQTN simulation scenario to 4.0% for the SQTN scenario compared to when 50 k SNPs were included in the prediction model (Table 5). We observed that the bias of the GEBV measured by regression slope was smallest when all QTN and the 50 k SNPs were included in the model (Table 6).

**Table 5:** Reliabilities of genomic prediction for one random replicate in each simulation scenario and different strategies for selection of rare and low-frequency variants (RLFV)

Scenarios	SQTN	MQTN	LQTN
50 k	35.7	43.0	38.0
50 k + all simulated QTN	42.5	46.4	43.6
50 k + simulated QTN and RLFV from 10 random selected genes from each chromosome	41.3	44.1	37.9
50 k + RLFV in mapped genes	39.7	44.3	40.1

SQTN corresponds to the scenario with RLFV in seven to ten genes per chromosome simulated as causal variants; MQTN corresponds to the scenario with RLFV in one gene per chromosome simulated as causal variants; LQTN corresponds to the scenario with RLFV in nine randomly selected genes across the whole genome simulated as causal variants. Reliabilities are presented on a scale from 0 to 100%. Accuracies, i.e. the square roots of the reliabilities, ranged from 0.597 to 0.681 on a scale from 0 to 1, while the corresponding standard errors ranged from 0.010 to 0.012

**Table 6:** Bias of the GEBV measured by regression slope for one random replicate in each simulation scenario and different strategies for selection of rare and low-frequency variants (RLFV)

Scenarios	SQTN	MQTN	LQTN
50 k	0.929	0.986	0.927
50 k + all simulated QTN	1.009	1.010	0.963
50 k + simulated QTN and RLFV from 10 random selected genes from each chromosome	0.995	1.016	0.938
50 k + RLFV in mapped genes	0.986	1.013	0.967

SQTN corresponds to the scenario with RLFV in seven to ten genes per chromosome simulated as causal variants; MQTN corresponds to the scenario with RLFV in one gene per chromosome simulated as causal variants; LQTN corresponds to the scenario with RLFV in nine randomly selected genes across the whole genome simulated as causal variants

Similar to the observed increase in prediction reliability when all simulated QTN and the 50 k SNPs were included for one replicate, the average prediction reliabilities improved from 1.9% for the SQTN scenario to 4.3% for the MQTN scenario across 10 replicates of each simulation scenario (see Additional file 3: Table S3). When randomly selected genic RLFV (without a simulated effect) were added to the simulated QTN, reliabilities decreased from 1.9% for the SQTN scenario to 3.0% for the MQTN scenario compared to when only simulated QTN (see Additional file 3: Table S3). Moreover, we observed that the bias of the GEBV measured by regression slope averaged across the 10 replicates decreased for all simulation scenarios when genic RLFV were added in the prediction model regardless of how they were selected (see Additional file 4: Table S4).

## Discussion

Earlier studies showed that using all imputed whole-genome sequence variants did not improve the reliability of genomic prediction [8], but it increased when using QTL derived from whole-genome sequence data [7, 12, 38]. Specifically, our aim was to examine empirically the impact of using selected RLFV from imputed whole-genome sequence data on the reliability of genomic prediction. We expected an improvement in the reliability of genomic prediction for the fitness-related traits fertility, health and longevity index by including imputed RLFV that jointly explained 13.3 to 24.6% of the explained DRP variance for these index traits in Nordic Holsteins [20]. Across the selection strategies applied here, the RLFV explained 4.0 to 31.1% of the explained DRP variance, but this was not reflected by changes in genomic prediction reliabilities, which varied by − 2.2 to 1.1%. This result is in line with the observation that the 50 k SNPs are able to capture most of the variance explained by the RLFV (Table 2).

This lack of improvement, and even decrease, in prediction reliabilities when using such selection strategies may be due to differences in imputation accuracy for the different selected subsets. An average imputation accuracy of 0.79 was found for the high impact annotation variants and of 0.81 for medium-to-high impact annotation variants, both these values being lower than the average value for all RLFV (0.85). However, it is unlikely that the decrease in prediction reliability using the high compared to the medium-to-high impact annotated RLFV is due to either this small difference in imputation accuracy or to a difference in MAF (the MAF for high impact annotation variants was on average 0.0162 and the MAF for medium-to-high impact variants was on average 0.0166). The main difference between the medium-to-high and high impact variants, is that the former group contained ~ 19 times more variants compared to the latter group. The fact that the reliabilities achieved with the medium-to-high impact variants were closer to those obtained in the scenario using all genic RLFV suggests that the benefit of this scenario may be due simply to the inclusion of many more RLFV. This is also in line with results of Hayes et al. [39] and Do et al. [40].

Low MAF levels imply that RLFV are only observed in a relatively small number of individuals, and some RLFV may be segregating only in either the training or validation population. Thus, such RLFV may contribute only slightly to the predictive ability at the population level. However, when we investigated this issue, we found that, in all cases, more than 92% of all genic RLFV were segregating in both the training and validation populations (see Additional file 5: Table S5). Thus, although the RLFV may be family-specific, in our data, they did have the potential to contribute to the predictive reliability.

Previous studies also showed that using sequence variants or preselected sequence variants, regardless of their MAF, yielded no or only marginal improvements in the accuracy of genomic prediction in dairy cattle [19, 41–43]. Related to this, Caballero et al. [44] showed that rare variants contribute little genetic variation to the phenotypic variation and are difficult to detect by GWAS. In the latter study, simulations were used to show that the common mutations of large effects are responsible for most of the genetic variation for quantitative traits except fitness [44]. However, we did observe that the bias of the GEBV measured by regression slope was slightly reduced when adding selected RLFV regardless of how they were selected for the health and longevity traits (Table 3) and also for the simulated datasets (see Additional file 4: Table S4). This result was also in line with the findings in [41, 43].

The selection scenario based on association mapping to identify the genic RLFV to be included in the prediction model, did not outperform predictions based on the 50 k data, in the analyses of the empirical data. This apparently low power of the selection scenario based on association mapping may have several causes. We used composite indices as phenotypes and these indices are a combination of several traits, and therefore the power to detect rare variants may be lower than for “ordinary” traits. Even if there was sufficient power to detect rare variants, their contribution in the prediction of composite indices may have been diluted in the index. As previously mentioned, using imputed rather than actual sequence data also reduces power. Moreover, the power to identify rare variants is low unless these variants or the combined RLFV in a gene or region have a large effect and a large sample size is used. For example, for a rare QTL that explains 0.1% of the total genetic variance, the power is not sufficient to identify it with the existing sample sizes of e.g. 5000 in dairy cattle [14]. In human studies, a very large sample size was used to detect associations of rare variants with phenotypes or diseases such as 60,564 individuals used for association mapping of rare variants with type 2 diabetes [45–47]. In our study, only ~ 5000 cattle individuals were available. It should be noted that the phenotypes used in this study are DRP based on large progeny groups, which have a minimal residual variance, and thus provide much stronger detection power than the own phenotypes used in human studies. Meanwhile, the number of detected genes containing RLFV was relatively large (see Additional file 1: Table S1), which suggests that it includes many false positives. Thus, the power to detect rare QTL for the real data of health-related traits was relatively low, and consequently adding selected RLFV in the prediction models did not only add extra information, but also noise. Previously, Perez-Enciso et al. [17] showed that the potential benefit of including causal loci in genomic prediction quickly disappears when there are too many incorrectly identified loci. This is supported by the results from our simulation scenarios that showed a small gain in reliability from using the mapped genic RLFV. Finally, the common SNPs genotyped on the 50 k array are, apparently, able to explain part of the trait variance due to the genic RLFV, leaving little room for improvement when adding genic RLFV to the model. The small effective population size in dairy cattle results in long range linkage disequilibrium (LD) across the whole genome and the common variants may be able to capture part of the effect from the genic RLFV due to co-segregation of QTL and marker alleles because of close family relationships [48].

We used a simulation to study these issues with selected RLFV in genomic prediction. Our results confirmed that adding non-causal RLFV to causal RLFV in the prediction model, indeed adds noise and considerably reduces the predictive ability. When significant genic RLFV from gene mapping were selected in the prediction model, the estimated variance component for genic RLFV was similar to the variance explained by the simulated QTN for all scenarios, in line with the improvement in predictive ability (see Additional file 6: Table S6). However, inclusion of the simulated QTN in the prediction model resulted in a larger improvement of the reliability compared to when significant genic RLFV from gene mapping were used (Table 5). These results suggest that improvement in prediction reliability can be achieved, provided that data with sufficient power is available to identify the causal RLFV affecting complex traits.

## Conclusions

We compared genomic prediction reliabilities using either only 50 k SNPs, or both 50 k SNPs and genic RLFV that were selected based on their association with the index trait studied or annotations of RLFV. Using either a selected subset of RLFV or all the genic RLFV, at best, marginally improved the reliability of genomic prediction, but decreased it in several cases (the change in reliability ranged from − 2.2 to 1.1%). Several reasons can explain these results, including low imputation accuracies for RLFV, a limited power to map genic RLFV with the existing sample size, and the fact that common variants on the 50 k chip are able to capture a large proportion of variance due to genic RLFV. However, we showed by using simulations that prediction reliability increased slightly when significant genic RLFV from association mapping were included in the prediction model and increased substantially when the known rare QTN were added as a separate genetic component in the model, but this added benefit was much reduced when adding additional random genic RLFV. This indicates that prediction reliability can be improved by using both 50 k data and selected genic RLFV, provided that the RLFV that explain a sizable variance in the index traits of interest are identified, without adding too many false positives that cause noise in the model.

## Additional files

**Additional file 1: Table S1.** Number of rare and low-frequency variants (RLFV) selected for inclusion in the genomic prediction model.

**Additional file 2: Table S2.** Characteristics for each simulation scenario across 10 replicates.

**Additional file 3: Table S3.** Reliabilities of genomic prediction using various marker sets for the index traits affected by different rare QTN sets, averaged over 10 replicates.^a^

**Additional file 4: Table S4.** Bias of the GEBV measured by regression slope using various marker sets for the index trait affected by different rare QTN sets, averaged over 10 replicates.

**Additional file 5: Table S5.** Proportion of rare or low-frequency variants (RLFV)^a^ segregating both in the validation and training populations from different models using various marker sets for fertility, health and longevity.

**Additional file 6: Table S6.** The additive genetic variances explained in the models for one replicate (the same replicate as selected for Tables 4, 5 and 6) in each simulation scenario and different strategies for selection of rare and low-frequency variants (RLFV).
