# Supplementary material for: Impact of rare and low-frequency sequence variants on reliability of genomic prediction in dairy cattle
Source: Genet Sel Evol. 2018 Nov 20;50:62. doi: 10.1186/s12711-018-0432-8 (PMC6247626; doi:10.1186/s12711-018-0432-8)
Supplement: Supplementary file 1 — Additional file 1: Table S1. Number of rare and low-frequency variants (RLFV) selected for inclusion in the genomic prediction model. [file 12711_2018_432_MOESM1_ESM.docx]

**Additional file 1 Table S1**

Format: docx

Title: Number of rare and low-frequency variants (RLFV) selected for inclusion in the genomic prediction model

| **Characteristics** | **Fertility** | **Health** | **Longevity** |
| --- | --- | --- | --- |
| The genic RLFV^a^ | 1,585,116 | 1,605,553 | 1,598,760 |
| RLFV in genes with significant association (number of genes with significant association) | 81,010 (1199) | 95,322 (1112) | 234,940 (2718) |
| RLFV with medium-to-high impact annotations^1^ | 495,516 | 529,341 | 545,370 |
| RLFV with high impact annotations^1^ | 25,944 | 27,619 | 28,620 |

^a^These numbers were different across index traits, because the RLFVs were extracted based on the individuals with phenotypes which differed across the three index traits (5,043, 4,926 and 4,673 for fertility, health and longevity respectively). The total number of RLFVs across the whole genome is 19,755,832.
