# Supplementary material for: Impact of rare and low-frequency sequence variants on reliability of genomic prediction in dairy cattle
Source: Genet Sel Evol. 2018 Nov 20;50:62. doi: 10.1186/s12711-018-0432-8 (PMC6247626; doi:10.1186/s12711-018-0432-8)
Supplement: Supplementary file 2 — Additional file 2: Table S2. Characteristics for each simulation scenario across 10 replicates. [file 12711_2018_432_MOESM2_ESM.docx]

**Additional file 2 Table S2**

Format: docx

Title: Characteristics for each simulation scenario across 10 replicates.

Description: The results are presented as mean (standard deviation). RLFV refers to rare and low-frequency variants and QTN refers to quantitative trait nucleotides. Scenario SQTN corresponds to the scenario with RLFV in seven to ten genes per chromosome simulated as causal variants; Scenario MQTN corresponds to the scenario with RLFV in one gene per chromosome simulated as causal variants; Scenario LQTN corresponds to the scenario with RLFV in nine randomly selected genes across the whole genome simulated as causal variants. The simulated total variances for the QTN in SQTN, MQTN and LQTN were 10% of the estimate of variance explained by 50k markers for fertility index.

| **Scenarios** | **SQTN** | **MQTN** | **LQTN** |
| --- | --- | --- | --- |
| Number of genes | 7 to10 per chr | 1 per chr | 9 |
| Number of SNPs on 50k array | 54,323 | 54,323 | 54,323 |
| Number of RLFV simulated as QTN | 22,212 (4359) | 1812 (192) | 523 (77) |
| Number of RLFV in the genes simulated as QTN and RLFV from 10 random selected genes from each chromosome | 46,036 (5177) | 27,445 (1367) | 26,729 (1171) |
