# Supplementary material for: Impact of rare and low-frequency sequence variants on reliability of genomic prediction in dairy cattle
Source: Genet Sel Evol. 2018 Nov 20;50:62. doi: 10.1186/s12711-018-0432-8 (PMC6247626; doi:10.1186/s12711-018-0432-8)
Supplement: Supplementary file 3 — Additional file 3: Table S3. Reliabilities of genomic prediction using various marker sets for the index traits affected by different rare QTN sets, averaged over 10 replicates.a [file 12711_2018_432_MOESM3_ESM.docx]

**Additional file 3 Table S3**

Format: docx

Title: Reliabilities of genomic prediction using various marker sets for the index traits affected by different rare QTN sets, averaged over 10 replicates^a^.

Description: The results are presented as mean (standard error). RLFV refers to rare and low-frequency variants and QTN refers to quantitative trait nucleotides. Scenario SQTN corresponds to the scenario with RLFV in seven to ten genes per chromosome simulated as causal variants; Scenario MQTN corresponds to the scenario with RLFV in one gene per chromosome simulated as causal variants; Scenario LQTN corresponds to the scenario with RLFV in nine randomly selected genes across the whole genome simulated as causal variants. The simulated total variances for the QTN in SQTN, MQTN and LQTN were equal to 10% of the estimated variance explained by 50k markers for the fertility index.

| **Scenarios** | **SQTN** | **MQTN** | **LQTN** |
| --- | --- | --- | --- |
| 50k | 39.4 (0.8) | 40.3 (0.5) | 39.4 (0.5) |
| 50k + simulated QTN | 41.3 (0.7) | 44.6 (0.5) | 42.9 (0.7) |
| 50k + simulated QTN and RLFV from 10 random selected genes from each chromosome | 40.4 (0.9) | 41.6 (0.6) | 40.8 (0.8) |

^a^Reliabilities and standard errors are presented on a scale from 0 to 100%.
