# Supplementary material for: Impact of rare and low-frequency sequence variants on reliability of genomic prediction in dairy cattle
Source: Genet Sel Evol. 2018 Nov 20;50:62. doi: 10.1186/s12711-018-0432-8 (PMC6247626; doi:10.1186/s12711-018-0432-8)
Supplement: Supplementary file 5 — Additional file 5: Table S5. Proportion of rare or low-frequency variants (RLFV)a segregating both in the validation and training populations from different models using various marker sets for fertility, health and longevity. [file 12711_2018_432_MOESM5_ESM.docx]

**Additional file 5 Table S5**

Format: docx

Title: Proportion of rare or low-frequency variants (RLFV)^a^ segregating both in the validation and training populations from different models using various marker sets for fertility, health and longevity

| **Marker sets** | **Fertility** | **Health** | **Longevity** |
| --- | --- | --- | --- |
| 50k SNP array | - | - | - |
| 50k + All genic RLFV | 94.22% | 92.90% | 94.48% |
| 50k + RLFV in genes with significant association | 95.27% | 93.86% | 94.95% |
| 50k + RLFV with medium-to-high impact annotations | 94.06% | 92.87% | 94.55% |
| 50k + RLFV with high impact annotations | 94.11% | 93.14% | 94.34% |

^a^The number of RLFV segregating both in the validation and training population was calculated as the count of rare or low-frequency heterozygotes or both common homozygotes and rare or low-frequency homozygotes in the validation and training populations.
