# Supplementary material for: Impact of rare and low-frequency sequence variants on reliability of genomic prediction in dairy cattle
Source: Genet Sel Evol. 2018 Nov 20;50:62. doi: 10.1186/s12711-018-0432-8 (PMC6247626; doi:10.1186/s12711-018-0432-8)
Supplement: Supplementary file 6 — Additional file 6: Table S6. The additive genetic variances explained in the models for one replicate (the same replicate as selected for Tables 4, 5 and 6) in each simulation scenario and different strategies for selection of rare and low-frequency variants (RLFV). [file 12711_2018_432_MOESM6_ESM.docx]

**Additional file 6 Table S6**

Format: docx

Title: The additive genetic variances explained in the models for one replicate (the same replicate as selected for Tables 4, 5 and 6) in each simulation scenario and different strategies for selection of rare and low-frequency variants (RLFV)

Description: SQTN corresponds to the scenario with RLFV in seven to ten genes per chromosome simulated as causal variants; MQTN corresponds to the scenario with RLFV in one gene per chromosome simulated as causal variants; LQTN corresponds to the scenario with RLFV in nine randomly selected genes across the whole genome simulated as causal variants. The simulated total variances for the QTN in SQTN, MQTN and LQTN were equal to 10% of the estimated variance explained by 50k markers for the fertility index.

| **Scenarios** | **SQTN** | | **MQTN** | | **LQTN** | |
| --- | --- | --- | --- | --- | --- | --- |
|  | 50k | RLFV | 50k | RLFV | 50k | RLFV |
| Variance simulated | 143.5 | 14.3 | 143.5 | 14.3 | 143.5 | 14.3 |
| 50k | 157.1 | - | 161.3 | - | 156.0 | - |
| 50k + All simulated QTN | 141.4 | 14.2 | 142.4 | 18.0 | 142.1 | 20.4 |
| 50k + RLFV in the genes simulated as QTN and RLFV from 10 random selected genes from each chromosome | 138.6 | 15.9 | 149.7 | 13.3 | 145.6 | 9.9 |
| 50k + RLFV in mapped genes | 154.3 | 14.7 | 141.6 | 18.2 | 148.7 | 19.3 |
